# Remote ischemic preconditioning prevents high‐altitude cerebral edema by enhancing glucose metabolic reprogramming

**DOI:** 10.1111/cns.70026

**Published:** 2024-09-02

**Authors:** Rongrong Han, Xiaoyan Yang, Xunming Ji, Bing Zhou

**Affiliations:** ^1^ Beijing Advanced Innovation Center for Big Data‐Based Precision Medicine Beihang University Beijing China; ^2^ China‐America Institute of Neuroscience, Xuanwu Hospital Capital Medical University Beijing China; ^3^ School of Medical Science and Engineering Beihang University Beijing China

**Keywords:** high‐altitude cerebral edema, hypoxia, metabolic reprogramming, mitochondrial dysfunction, remote ischemic preconditioning

## Abstract

**Aims:**

Incidence of acute mountain sickness (AMS) ranges from 40%–90%, with high‐altitude cerebral edema (HACE) representing a life‐threatening end stage of severe AMS. However, practical and convenient preventive strategies for HACE are lacking. Remote ischemic preconditioning (RIPC) has demonstrated preventive effects on ischemia‐ or hypoxia‐induced cardiovascular and cerebrovascular diseases. This study aimed to investigate the potential molecular mechanism of HACE and the application of RIPC in preventing HACE onset.

**Methods:**

A hypobaric hypoxia chamber was used to simulate a high‐altitude environment of 7000 meters. Metabolomics and metabolic flux analysis were employed to assay metabolite levels. Transcriptomics and quantitative real‐time PCR (q‐PCR) were used to investigate gene expression levels. Immunofluorescence staining was performed on neurons to label cellular proteins. The fluorescent probes Mito‐Dendra2, iATPSnFR1.0, and CMTMRos were used to observe mitochondria, ATP, and membrane potential in cultured neurons, respectively. TUNEL staining was performed to detect and quantify apoptotic cell death. Hematoxylin and eosin (H&E) staining was utilized to analyze pathological changes, such as tissue swelling in cerebral cortex samples. The Rotarod test was performed to assess motor coordination and balance in rats. Oxygen–glucose deprivation (OGD) of cultured cells was employed as an in vitro model to simulate the hypoxia and hypoglycemia induced by RIPC in animal experiments.

**Results:**

We revealed a causative perturbation of glucose metabolism in the brain preceding cerebral edema. Ischemic preconditioning treatment significantly reprograms glucose metabolism, ameliorating cell apoptosis and hypoxia‐induced energy deprivation. Notably, ischemic preconditioning improves mitochondrial membrane potential and ATP production through enhanced glucose‐coupled mitochondrial metabolism. In vivo studies confirm that RIPC alleviates cerebral edema, reduces cell apoptosis induced by high‐altitude hypoxia, and improves motor dysfunction resulting from cerebral edema.

**Conclusions:**

Our study elucidates the metabolic basis of HACE pathogenesis. This study provides a new strategy for preventing HACE that RIPC reduces brain edema through reprogramming metabolism, highlighting the potential of targeting metabolic reprogramming for neuroprotective interventions in neurological diseases caused by ischemia or hypoxia.

## Graphical abstract

RIPC ameliorated brain edema induced by high‐altitude hypoxia through stimulating glucose metabolism down to the TCA cycle to boost mitochondrial ATP production. This finding supports the potential of RIPC as a viable therapeutic approach for the prevention and treatment of HACE, targeting metabolic reprogramming. This figure was created with BioRender (https://www.biorender.com).

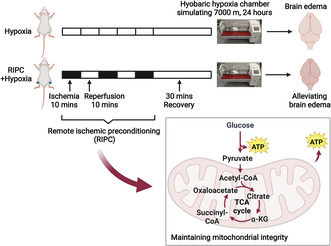

## 1 INTRODUCTION

Many people travel to high altitudes for various reasons such as pleasure, work, and athletic competitions. However, rapid ascents put them at risk of acute mountain sickness (AMS).^1^ The principal symptoms of AMS are headache, loss of appetite, nausea, vomiting, dizziness, fatigue, and insomnia.^2^ Living at high altitudes (>3500 meters) requires physiological adaptations to compensate for the lower partial pressure of oxygen. When the body is exposed to a plateau or other hypoxic environment, exemplified by a drop in oxygen partial pressure to 45 mmHg (approximately corresponding to an altitude of 4000 meters), severe cognitive impairment and truncal ataxia may occur.^2^ The prevalence of AMS ranges from 40%–90% in unacclimatized individuals ascending above 500 m per day to altitudes of 4500–6000 meters,^3^, ^4^ depending on altitude and individual maladaptation. High‐altitude cerebral edema (HACE), the severe and potentially life‐threatening stage of AMS, occurs in 0.5%–1% of elevations between 4200 and 5500 meters, increasing to 30%–50% at an altitude from 5500 to 8000 meters.^5^, ^6^ Once AMS occurs, untreated AMS can rapidly progress into HACE.^7^ Untreated HACE can lead to coma and death within 24 h.^1^ However, effective prevention and treatment to counteract hypoxia are limited by an incomplete understanding of the molecular mechanisms underlying HACE, especially the metabolic changes caused by hypoxemia. Our study aims to uncover the previously uncharacterized metabolic basis of HACE in brain tissues, offering insights into preventing HACE and potential therapeutic targets.

Due to its rapid progression and high mortality, the prevention of HACE should take priority over its treatment. The most widely recommended strategy for preventing AMS and HACE is a gradual ascent.^8^ However, modern transportation, such as planes and trains, enables people to rapidly ascend to high altitudes, leading climbers to abandon a gradual ascent and the opportunity to acclimate gradually. This acclimatization process is crucial for preventing AMS and HACE. Pharmacologic prevention of AMS,^8^ such as acetazolamide, dexamethasone, and ibuprofen, has the potential side effects and limitations: acetazolamide can cause paresthesia and reduce exercise capacity^9^, ^10^; dexamethasone can risk adrenal suppression^11^, ^12^; and the long‐term safety of ibuprofen (600 mg three times daily) is unproven.^13^, ^14^ Additionally, supplemental oxygen may help to relieve symptoms of HACE but has limited accessibility.^8^, ^15^ Therefore, new, effective, and safe prevention strategies for AMS and HACE are urgently needed.

Remote ischemic preconditioning (RIPC) is an intrinsic process whereby repeated short episodes of non‐lethal ischemia/reperfusion in double‐sided limbs can protect distant organs such as the brain, heart, and kidneys from lethal ischemic injury.^16^, ^17^, ^18^ The mechanisms underlying RIPC are intricate and remain to be fully elucidated. It is postulated that three principal pathways are activated: humoral pathway, neural pathway, and genetic/inflammatory pathway, which facilitate the transfer of remote stimuli to the target organs, thereby conferring protection.^19^, ^20^ Several studies in healthy volunteers found that RIPC intervention is associated with improved oxygen saturation and cardiac function, as well as attenuation of hypoxic pulmonary vasoconstriction following rapid ascent to high altitude (3650–4000 meters).^21^, ^22^, ^23^ It has also been observed to ameliorate spatial memory and sleep quality in high‐altitude hypoxic environments.^24^ It is conceivable that RIPC might also prevent AMS and HACE. The fundamental functions of neurons require a substantial amount of energy, primarily derived from an adequate oxygen supply to support mitochondrial oxidative phosphorylation. Under hypoxic conditions, increased glycolysis may partially fulfill energy demands. However, the modulation of these processes in HACE and their relationship with RIPC remain unclear. Our study aimed to elucidate the metabolic mechanisms underlying RIPC as an intervention for alleviating HACE in rats exposed to high‐altitude conditions. Understanding these metabolic mechanisms facilitates the development of new drugs targeting metabolism to prevent and treat HACE.

## 2 MATERIALS AND METHODS

### 2.1 Animals

Male Sprague–Dawley (SD) rats weighing 300 ± 10 g were purchased from Vital River Laboratories. The animals were maintained in a facility with 12 h light/dark cycle, free access to food and water, and controlled temperature and humidity at 23°C ± 2°C and 60% ± 5%, respectively. Animal research was conducted following the ARRIVE (Animal Research: Reporting of In Vivo Experiments) guidelines 2.0. All animal procedures were performed according to National Institutes of Health guidelines and were approved by the Animal Care and Use Committee of Beihang University, China.

### 2.2 In vivo experiments

#### 2.2.1 Remote ischemic preconditioning

Rats were randomly assigned to the control, hypoxia, and hypoxia‐RIPC groups. For the RIPC group, rats were anesthetized with sodium pentobarbital (30 mg/kg, i.p.), and then hind limb occlusion was accomplished by tightening a tourniquet (8 cm) around the upper thigh for three cycles, with each occlusion or release phase lasting 10 min. To exclude potential synergistic effects of anesthetics, the control and hypoxia groups received the same dosage of sodium pentobarbital without RIPC. During anesthesia with sodium pentobarbital, a heating pad maintained body temperature homeostasis in the rats.

#### 2.2.2 High‐altitude hypoxia treatment

For acute hypobaric hypoxia treatment, rats were placed in a decompression chamber (Fenglei) mimicking an altitude of 7000 meters. The chamber humidity was maintained at 40%–50%, and a continuous fresh air flow of 5 L/h was supplemented to avoid CO_2_ accumulation. The chamber temperature was maintained at 22–24°C to minimize the hypothermic effects of hypoxia on the rats. Rats from the hypoxia and hypoxia‐RIPC groups were exposed to acute hypobaric hypoxia with free access to food and water. Control rats under normoxic conditions were kept outside the chamber in the same laboratory location.

#### 2.2.3 Determination of brain water content

After exposure to high‐altitude hypoxic conditions, brain tissues were immediately collected from rats anesthetized with 1% (v/v) sodium pentobarbital intraperitoneally. The wet weight of each brain was determined using a precision electronic balance (Sartorius). To obtain a constant weight defined as the dry weight, the brain tissues were then placed in an electric‐thermostatic baking oven at 120°C for 24 h and reweighed. The percentage of brain water content was calculated as follows: water content (%) = (wet weight‐dry weight)/wet weight × 100%.

#### 2.2.4 Hematoxylin and eosin (H&E) staining

Tissue sections mounted on slides were initially deparaffinized in xylene for 5 min, followed by rehydration through a graded ethanol series (100%, 95%, 80%, 70%). After this preparation, the slides were immersed in hematoxylin solution for 10 min, differentiated in 1% acid alcohol, and rinsed in tap water for 5 min. Subsequently, counterstaining was performed with eosin Y for 5 min, followed by rinsing in distilled water to remove excess stain. The slides were then dehydrated in a series of ethanol concentrations, cleared in xylene, and mounted with coverslips for microscopic examination.

#### 2.2.5 Rotarod test

To assess the motor coordination of rats, the Rotarod test was performed 1 h after high‐altitude hypoxia exposure. Briefly, rats were trained for 4 days to acclimate to the Rotarod. After various treatments, the rats were placed on an accelerating Rotarod cylinder with the speed increasing from 4 to 40 rpm within 5 min. Each trial lasted for a maximum of 10 min and ended when rats fell off or gripped the device and spun around for two consecutive revolutions without trying to walk on the rungs.

### 2.3 Cell experiments

#### 2.3.1 Primary neuron culture

Primary cortical neurons were cultured as previously described.^25^ The isolated neuronal cell bodies were cultured at a final density of approximately 2000 cells per 12 mm coverslip. After 7–10 days of culture in vitro, neurons were treated and stained for imaging.

#### 2.3.2 Ischemic preconditioning (IPC) on neurons

IPC and hypoxia treatment were initiated using a specialized, humidified chamber kept at 37°C, containing an anaerobic gas mixture (94% N_2_, 1% O_2_, and 5% CO_2_). The gas tap was opened and the chamber was inflated for 5 min to expel oxygen, with the gas flow controlled at 20–30 L/min. For IPC, coverslips were transferred from the culture medium to oxygen–glucose deprivation (OGD) buffer, followed by 5 min of OGD and subsequent reperfusion for 30 min. During reperfusion, the original culture medium was transferred to a new dish, in which the coverslips were placed, and then incubated. Hypoxia treatment was performed in the same chamber immediately following reperfusion.

#### 2.3.3 Lentivirus infection

The mito‐Dendra2 and iATPSnFR (Addgene #102548) were cloned into the pFUGW vector. All lentiviral vectors were prepared and transduced at the same concentration. For infection, 5 μL of concentrated virus was added to 3 × 10^5^ freshly dissociated neurons in 2 mL of culture medium.

#### 2.3.4 ATP assessment

Cultured neurons infected with iATPSnFR were stained with MAP2 using immunofluorescence and imaged with high‐speed confocal microscopy (Andor Dragonfly 200). The ATP levels, indicated by the fluorescence intensity of iATPSnFR, were quantified using the ImageJ software (NIH).

#### 2.3.5 Measurement of mitochondria membrane potential (Δψm)

Following treatment, neurons were incubated with 100 nM MitoTracker™ Orange CMTMRos (Invitrogen) for 30 min at 37°C. Subsequently, the cells were washed with 1 × PBS and fixed with 4% paraformaldehyde. Neurons were further stained with MAP2. The Δψm was assessed by measuring the relative fluorescent intensity of the orange‐fluorescent dye in MAP2‐positive neurons using ImageJ particle analysis.

#### 2.3.6 Immunofluorescence

Cultured cortical neurons on coverslips were utilized for immunofluorescence studies. Following treatment, neurons were fixed with 4% paraformaldehyde in 1 × PHEM buffer (18.14 g PIPES, 6.5 g HEPES, 3.8 g EGTA, and 0.99 g MgSO_4_ in 1 L ddH_2_O, adjusted pH to 7.0), which is good for fixing mitochondria, and were blocked for 1 h at room temperature using a blocking buffer. Subsequently, they were incubated with primary antibodies in the same blocking buffer. The primary antibodies were anti‐MAP2 antibody (1:1000, Millipore) and anti‐TOM20 antibody (1:1000, Abcam). After overnight incubation with the mixed primary antibodies at 4°C, the neurons were incubated with corresponding secondary antibodies. The coverslips were then mounted with ProLong™ Glass Antifade Mountant with or without NucBlue™ Stain (Invitrogen). All images were captured using high‐speed confocal microscopy (Andor Dragonfly 200).

#### 2.3.7 TDT‐mediated dUTP nick end labeling (TUNEL)

TUNEL was performed following the standard protocols provided in the manual of the In Situ Cell Death Detection Kit (Cat. No. 11684817910, Roche). Neurons were subjected to three types of immunofluorescent staining, including the TUNEL assay, the MAP2 staining, and the nuclear DAPI (blue) staining. Subsequently, the ratio of cells exhibiting positive staining with green fluorescence was calculated.

### 2.4 Metabolomics and transcriptomics

#### 2.4.1 Collecting samples for metabolomics and transcriptomics

To ensure accuracy in metabolite analysis, all rats were euthanized via cervical dislocation in a chilled environment, avoiding disturbances to metabolite profiles that may result from alternative methods like CO_2_‐induced anesthesia. Immediately following euthanasia, perfusion with 50 mL of ice‐cold physiological saline was performed to rapidly cool the bodies on ice, within 2 min, to inhibit enzymatic activities that could alter metabolite levels. This perfusion also served to wash away blood metabolites, thereby minimizing their potential impact on brain tissue metabolite levels. After prompt brain extraction, the tissues were rapidly immersed in liquid nitrogen. The entire procedure on ice was completed within 5 min. For cultured cells, neurons were exposed to the medium containing ^13^C_6_‐U‐glucose (CLM‐1396‐5, Cambridge) for 4 h to label metabolites. After removal of the medium, the cells were washed with PBS and promptly flash‐frozen in liquid nitrogen.

#### 2.4.2 RNA extraction and RNA‐seq

Total RNA extraction was performed according to the manufacturer's protocol of the animal tissue RNA extraction kit (Tiangen). Briefly, cerebral cortex tissues stored in liquid nitrogen were thawed and transferred to a lysis buffer supplemented with β‐mercaptoethanol, followed by thorough homogenization. RNA was then immediately isolated, purified, and eluted. The extracted RNA samples were temporarily stored at −80°C until further analysis.

The integrity, purity, and concentration of RNA in each sample were assessed using the 5200 Fragment Analyzer (Agilent Technologies). Samples with an RNA Quality Number (RQN) exceeding 8.0 were selected for library preparation. Sequencing libraries were prepared and sequenced on the DNBSEQ platform by Shenzhen BGI Technology Co., Ltd. Initial quality checks (QC) were performed on the raw sequencing reads. Following QC, cleaned reads were mapped to the reference genome, and the alignment's efficacy was evaluated by analyzing the statistical mapping rate and the evenness of read coverage across the reference, ensuring the data met the prerequisites for secondary QC.

RNA‐seq analysis was conducted using the Dr. TOM2 system (BGI Technologies) to identify differentially expressed genes and perform enrichment analyses of Gene Ontology (GO) and Kyoto Encyclopedia of Genes and Genomes (KEGG) pathways. Additionally, the RNA‐seq results were validated using qPCR.

#### 2.4.3 Metabolic labeling and metabolome analysis

Cerebral cortex tissues or cultured cortical neurons were lysed with methanol and subsequently centrifuged at 14,000 *g* for 10 min at 4°C. The supernatant was transferred to new Eppendorf tubes and dried using a SpeedVac system (miVac, GeneVac). Post‐drying, all samples were briefly stored at −80°C until further analysis. For the liquid chromatography–mass spectrometry (LC–MS) assay, the dried samples were reconstituted into 100 μL of methanol: water (2:8, LC–MS grade, Fisher Scientific), and a 5 μL aliquot was subjected to LC–MS/MS analysis. To ensure the precision of metabolite measurements, standard samples were utilized as quantitative references during the metabolite detection process.

Multiple reaction monitoring (MRM) analysis used the QTRAP 6500, a hybrid triple quadrupole/linear ion trap (AB SCIEX). Metabolite data, including precursor‐product ion pairs and retention time confirmed by parallel reaction monitoring (PRM) results, were incorporated into a transition list for MRM assay (237 Q1/Q3 transitions for 237 metabolites). The MRM assay was performed in a positive–negative ion‐switching mode without scheduling. An Xbridge amide column (length: 100 mm; inner diameter: 4.6 mm; particle size: 3.5 μm; pore size: 130 Å, Waters) was employed for compound separation at 30°C.

Using the MRMPROBS software, metabolite data, encompassing metabolite names, peak intensities, and sample identifiers, were precisely extracted. Subsequent metabolomic analysis was conducted using MetaboAnalyst 5.0 (http://www.metaboanalyst.ca/MetaboAnalyst/faces/home.xhtml).

### 2.5 Quantitative real‐time PCR (qPCR)

Total RNA was extracted from the rat brain cortex using the RNA extraction kit (TIANGEN), followed by cDNA synthesis using Maxima H Minus cDNA Synthesis Master Mix with DNase (Thermo Fisher Scientific). Real‐time PCR was performed using FastStart Universal SYBR Green Master (Roche) to assess relative gene expression. A fragment of actin served as the internal control, and the primers utilized in this study are listed in Table S1. Differences in gene expression (DEG) were calculated using the 2^(−ΔΔCT)^ method and are presented as the relative fold change.

### 2.6 Quantification and statistical analysis of the data

The exact value of replicates (*n*) is indicated in the figure legends and refers to the number of biological replicates. There were no inclusion criteria, and no data were excluded. Statistical analysis was performed using GraphPad Prism 8.0. All data are represented as mean ± standard deviation (SD) unless otherwise specified. Before conducting statistical analyses, all data underwent normality testing using either the Shapiro–Wilk test or the Kolmogorov–Smirnov test. For normally distributed data with equal variances, comparisons between two groups were conducted using the two‐tailed unpaired *t*‐test. When dealing with more than two groups, one‐way ANOVA was employed, followed either by Dunnett's multiple comparisons test (for comparisons of all groups against a control group) or Tukey's multiple comparisons test (for comparisons among all groups). For data not adhering to a normal distribution, the two‐tailed Mann–Whitney test was applied for comparisons between two groups, and the Kruskal–Wallis test, followed by Dunnett's multiple comparisons test, was used for comparisons among more than two groups. A two‐tailed *p*‐value of less than 0.05 was considered to indicate statistical significance.

## 3 RESULTS

### 3.1 Glucose metabolic dysfunction in HACE

**FIGURE 1:**
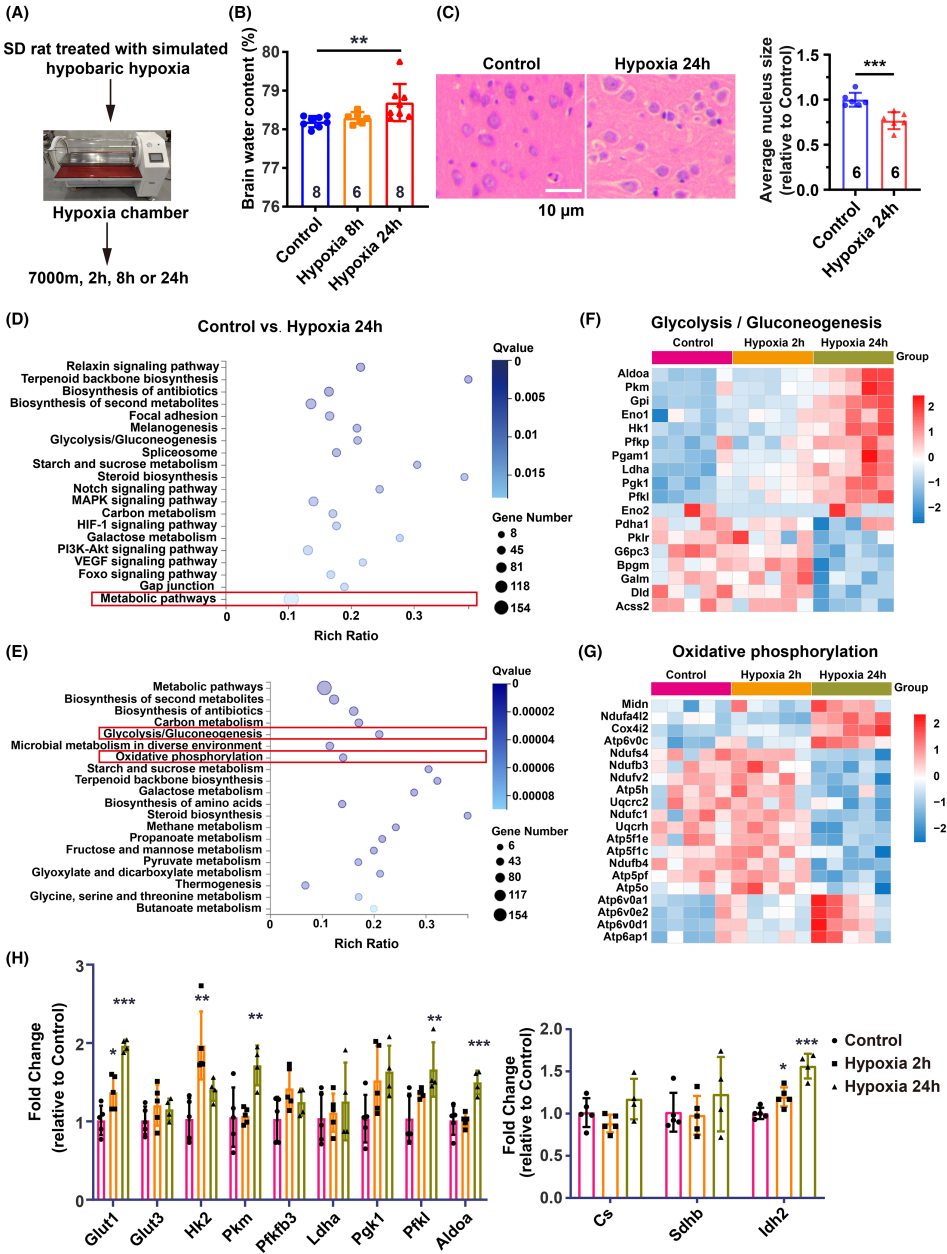
Disturbance of gene expression involved in glucose metabolism in the rat brain cortex induced by high‐altitude hypoxia. (A) Graphical diagram of high‐altitude hypoxia treatment. (B) Quantification of brain water content in SD rats, with *n* = 6 or 8 per group. (C) H&E staining of the brain cortex and quantification of cell nucleus area ratio showing brain edema. *n* = 6 fields per group, scale bar: 10 μm. (D) KEGG Pathway enrichment of significantly changed genes of Control versus Hypoxia 24 h obtained from RNA sequencing. (E) Pathway enrichment of genes in metabolic pathways obtained in (D). (F,G) Heatmap of significantly changed genes in Glycolysis/Gluconeogenesis and Oxidative phosphorylation pathways. (H) Verification of gene expression in glycolysis and the TCA cycle by real‐time PCR, with *n* = 4 or 5 per group. Data are presented as the mean ± SD. Statistical analysis was performed using the Shapiro–Wilk test for normality of all data; the Kruskal–Wallis test followed by Dunn's multiple comparisons test for (B) and for Hk2 and Pfkl in (H); one‐way ANOVA followed by Dunnett's multiple comparisons test for Glut1, Idh2, Pkm, and Aldoa in (H); and a two‐sided unpaired *t*‐test for (C). *p < 0.05, **p < 0.01, ***p < 0.001.

To induce brain edema through high‐altitude hypoxia, we placed Sprague–Dawley (SD) rats (300 g ± 10 g) in a hypobaric hypoxia chamber mimicking an altitude of 7000 meters and exposed them to hypoxia for 8 or 24 h, respectively (Figure 1A). After 8 h of hypoxia exposure, the brain water content of SD rats did not change significantly, whereas it significantly increased after 24 h hypoxia exposure (Figure 1B), indicating the occurrence of HACE following 24 h hypoxia exposure. Pathological analysis using H&E staining showed cell swelling in the cerebral cortex and significant enlargement of the intracellular space, as indicated by the ratio of the nucleus to the whole cell area, at 24 h post‐hypoxia treatment (Figure 1C).

To delineate the metabolic alterations following the initial hypoxia exposure and subsequent onset of cerebral edema, we conducted RNA sequencing and metabolomic analyses on the cerebral cortex tissue from control, 2 h hypoxia, and 24 h hypoxia groups (Figure 1A). Enrichment analysis via KEGG pathways revealed significant changes in the expression of 154 genes involved in metabolic processes (Figure 1D). Subsequent pathway clustering indicated notable differences in the expression of genes associated with glycolysis and oxidative phosphorylation within glucose metabolism‐related pathways (Figure 1E). Heatmap visualization demonstrated a significant upregulation of glycolysis‐related genes following 24 h of hypoxia, in contrast to a marked downregulation of genes linked to oxidative phosphorylation (Figure 1F,G). Real‐time PCR confirmed increased expression of genes responsible for glucose transport (Glut1) and pivotal glycolytic enzymes (Hk2, Pkm, Pgk1, Pfkl, and Aldoa) after 24 h of hypoxia exposure (Figure 1H). Conversely, the expression levels of genes governing tricarboxylic acid (TCA) cycle enzymes remained relatively unchanged or exhibited a slight increase (Idh2), potentially reflecting negative feedback regulation due to the suppression of oxidative phosphorylation (Figure 1H).

**FIGURE 2:**
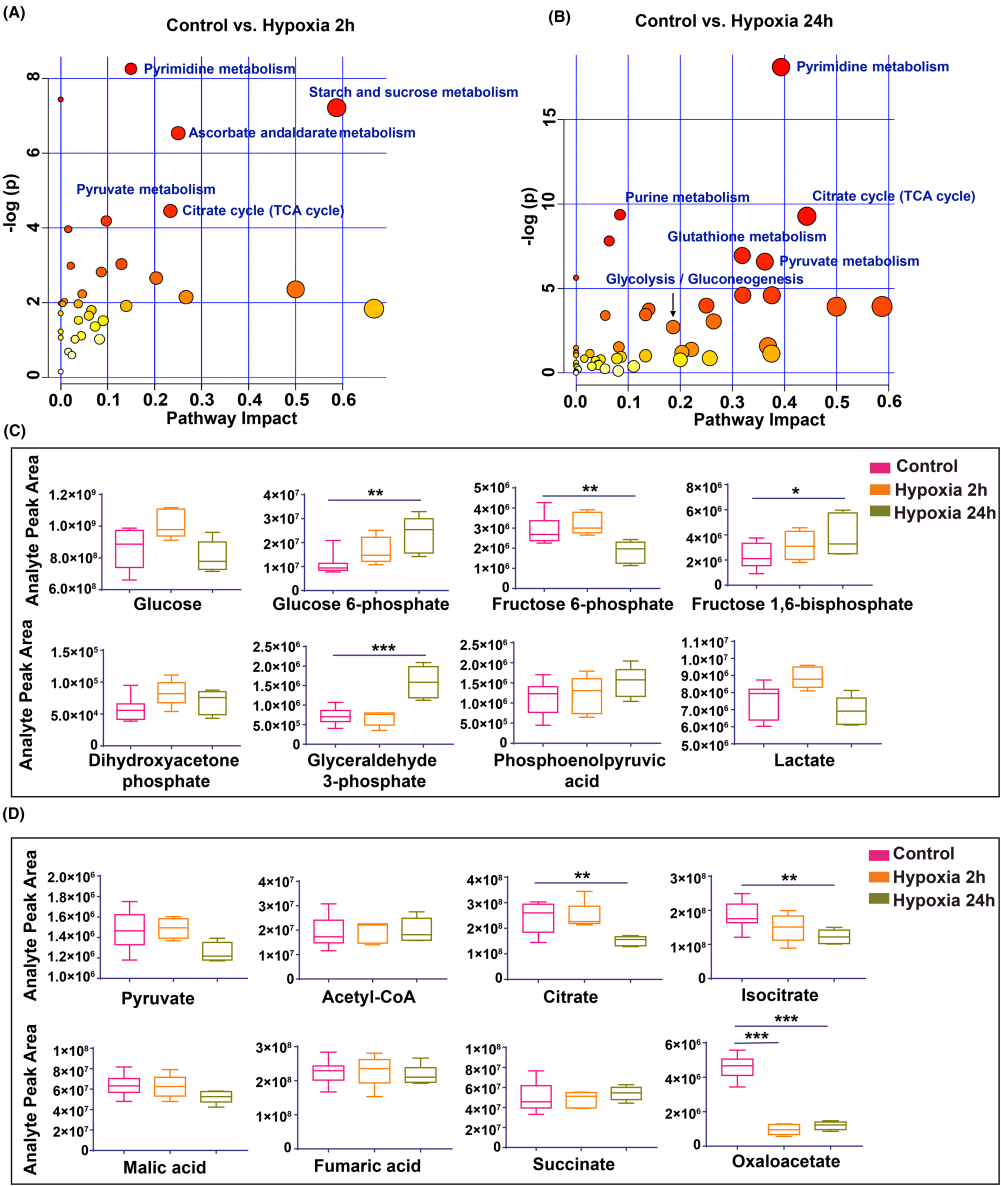
Glucose metabolic disturbance in the rat brain cortex induced by high‐altitude hypoxia. (A) Pathway enrichment of significantly changed metabolites in Control versus Hypoxia 2 h. The color indicates the value of −log(P), and the size of the dots represents the pathway impact. (B) Pathway enrichment of significantly changed metabolites in Control versus Hypoxia 24 h. The color represents the value of −log(p), while the size of the dots indicates the pathway impact. (C,D) Changes in metabolites involved in glycolysis and the TCA cycle. Data are expressed as min to max, with *n* = 5–6 for each group. Statistical analysis was performed using the Shapiro–Wilk test or the Kolmogorov–Smirnov test for normality, followed by one‐way ANOVA and Dunnett's multiple comparisons test. *p < 0.05, **p < 0.01, ***p < 0.001.

To elucidate the correlation between metabolic gene expression changes and metabolite levels, we conducted a metabolomic analysis using LC–MS. Pathway enrichment analysis revealed significant alterations in metabolites associated with the TCA cycle and pyruvate metabolism after 2 h of hypoxia (Figure 2A). After 24 h of hypoxia, the significantly altered metabolites were predominantly involved in glucose metabolism pathways, including glycolysis, pyruvate metabolism, and the TCA cycle (Figure 2B).

The early hypoxic response at 2 h was characterized by elevated glucose and lactate levels, along with increased glycolytic intermediates (Figure 2C), indicative of an enhanced glycolytic response that surpasses mitochondrial metabolic capacity. In contrast, after 24 h of hypoxia, glucose levels declined, while glycolytic intermediates such as glucose 6‐phosphate, fructose 1,6‐bisphosphate, and glyceraldehyde 3‐phosphate accumulated. Concurrently, lactate, the end product of glycolysis, was reduced, suggesting glycolytic inhibition after prolonged hypoxia (Figure 2C), potentially indicating mitochondrial damage or decoupling of glycolysis from mitochondrial metabolism.

To further investigate mitochondrial metabolic activity, we scrutinized TCA cycle metabolites. We found that, with the exception of oxaloacetate, which decreased in the hypoxia group due to impaired NADH oxidation, most TCA intermediates did not exhibit significant changes in the 2 h exposure group (Figure 2D). This suggests that while TCA cycle fluxes are disrupted, metabolite transport from the cytosol to the mitochondria remains active.^26^ Notably, after 24 h of hypoxia, there was a subsequent decrease in citrate, isocitrate, malic acid, and oxaloacetate levels, underscoring limited regenerative mechanisms due to insufficient pyruvate oxidation and compromised mitochondrial oxidative phosphorylation.

These findings imply that the coupling of glucose metabolism to mitochondrial function is disrupted following 24 h of hypoxia. Furthermore, the onset of these metabolic changes can be traced to as early as 2 h into the hypoxic treatment, potentially affecting energy production.

### 3.2 Decreased neuronal ATP and mitochondrial dysfunction in HACE

**FIGURE 3:**
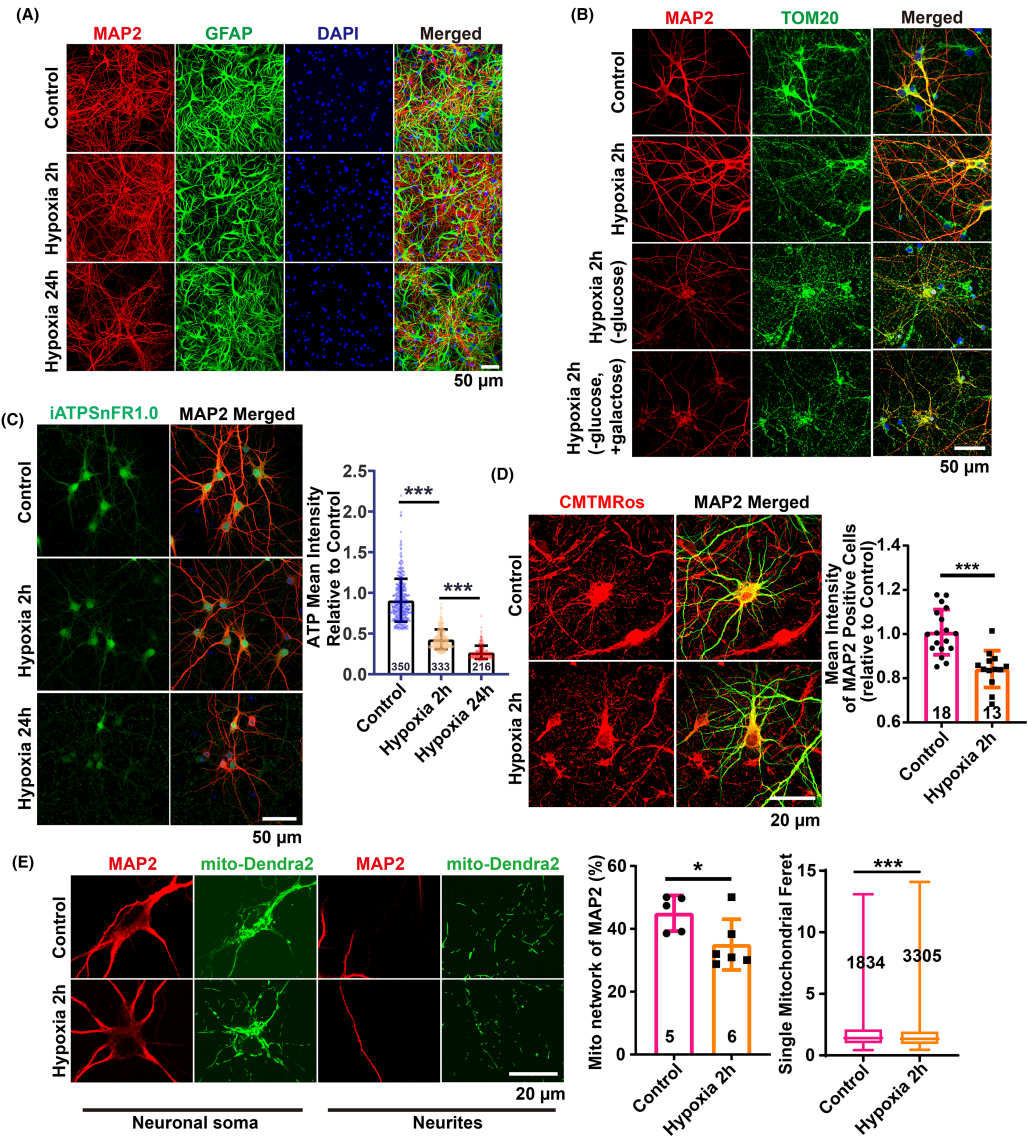
Decreased ATP and mitochondrial dysfunction in primary cortical neurons induced by hypoxia. (A) Images showing the morphological changes of MAP2‐labeled neurons and GFAP‐labeled astrocytes after hypoxia treatment. (B) Images showing the morphological changes of neurons labeled by MAP2 and mitochondria labeled by TOM20 induced by hypoxia in culture medium with or without glucose deprivation. (C) Images and quantitative analysis showing significantly decreased intracellular ATP labeled by the iATPSnFR1.0 vector post‐hypoxia treatment. (D) Images and quantitative analysis showing significantly decreased mitochondrial membrane potential indicated by MitoTracker™ Orange CMTMRos after hypoxia (1% O_2_) treatment for 2 h. (E) Images and quantitative analysis showing the decreased mitochondrial network in neuronal soma and increased fragmentation of mitochondria in neurites after 2 h of hypoxia treatment. Scale bars: 50 μm (A–C), 20 μm (D,E). All data are presented as mean ± SD, with *n* = 3 biological replicates, and the total number of images for analysis is indicated within the bars. Statistical analysis was performed using the Shapiro–Wilk test for normality of (C,D) and the mitochondrial Feret diameter in (E); the Kolmogorov–Smirnov test for normality of the mitochondrial network in (E); the Kruskal–Wallis test followed by Dunnett's multiple comparisons test for (C); a two‐sided unpaired *t*‐test for (D) and for the mitochondrial network in (E); and the Mann–Whitney test for the mitochondrial Feret diameter in (E). *p < 0.05, ***p < 0.001.

Disturbed energy supply is a critical contributor to the pathogenesis of brain edema. To ascertain whether hypoxic conditions at high altitudes precipitate disturbances in glucose metabolism and concomitantly lead to inadequate cellular energy, we sought direct in vitro evidence. We employed oxygen deprivation (1% O_2_) to mimic the effects of high‐altitude hypoxia on cells. A co‐culture system of primary rat cortical neurons and astrocytes was subjected to 1% O_2_ for 24 h. Immunofluorescence staining for neuronal and astrocytic markers revealed rosary‐like disruptions in neuronal integrity, while astrocytes maintained their normal morphology (Figure 3A). This suggests an increased vulnerability of neurons to hypoxia, underscoring the need to prioritize neuronal protection under such conditions.

Neurons at 7 days in vitro (DIV7) exposed to 1% O_2_ for 2 h displayed no significant morphological alterations upon MAP2 staining. However, neuronal and mitochondrial integrity were compromised when glucose was omitted from the culture medium or replaced with galactose, which inhibits glycolysis through competing with glucose for phosphorylation by hexokinase, during hypoxia (Figure 3B). This indicates that glucose metabolism is vital for preserving neuronal structure and function under hypoxic stress.

To examine whether a disruption in glucose metabolism under hypoxia results in diminished ATP production, which other metabolic substrates may compensate for, we quantified intracellular total ATP levels using a lentivirus‐packaged ATP‐targeting vector, iATPSnFR1.0.^27^ Notably, hypoxic treatments of 1% O_2_ for both 2 and 24 h led to a significant reduction in neuronal ATP (Figure 3C). Considering that aerobic glucose metabolism in mitochondria is essential for ATP synthesis via oxidative phosphorylation, the observed ATP decrease is indicative of mitochondrial dysfunction.

Consistently, after 2 h of hypoxia, mitochondrial morphological abnormalities were evident from TOM20 staining (Figure 3B), and MitoTracker™ Orange CMTMRos staining revealed a substantial decline in the mitochondrial membrane potential in MAP2‐specific neurons (Figure 3D). Notably, although the mitochondrial networking consistently decreased and fragmentation was observed using mito‐dendra2 labeling, neuron integrity was preserved. This suggests a compensatory role of enhanced glycolysis in ATP production to maintain the cytoskeletal integrity during 2 h of hypoxic exposure (Figures 2C and 3B–E). Collectively, these findings implicate hypoxia‐induced mitochondrial damage as a causative factor for subsequent disruptions in neuronal integrity.

### 3.3 Ischemic preconditioning (IPC) plays a protective role on neurons during hypoxia

**FIGURE 4:**
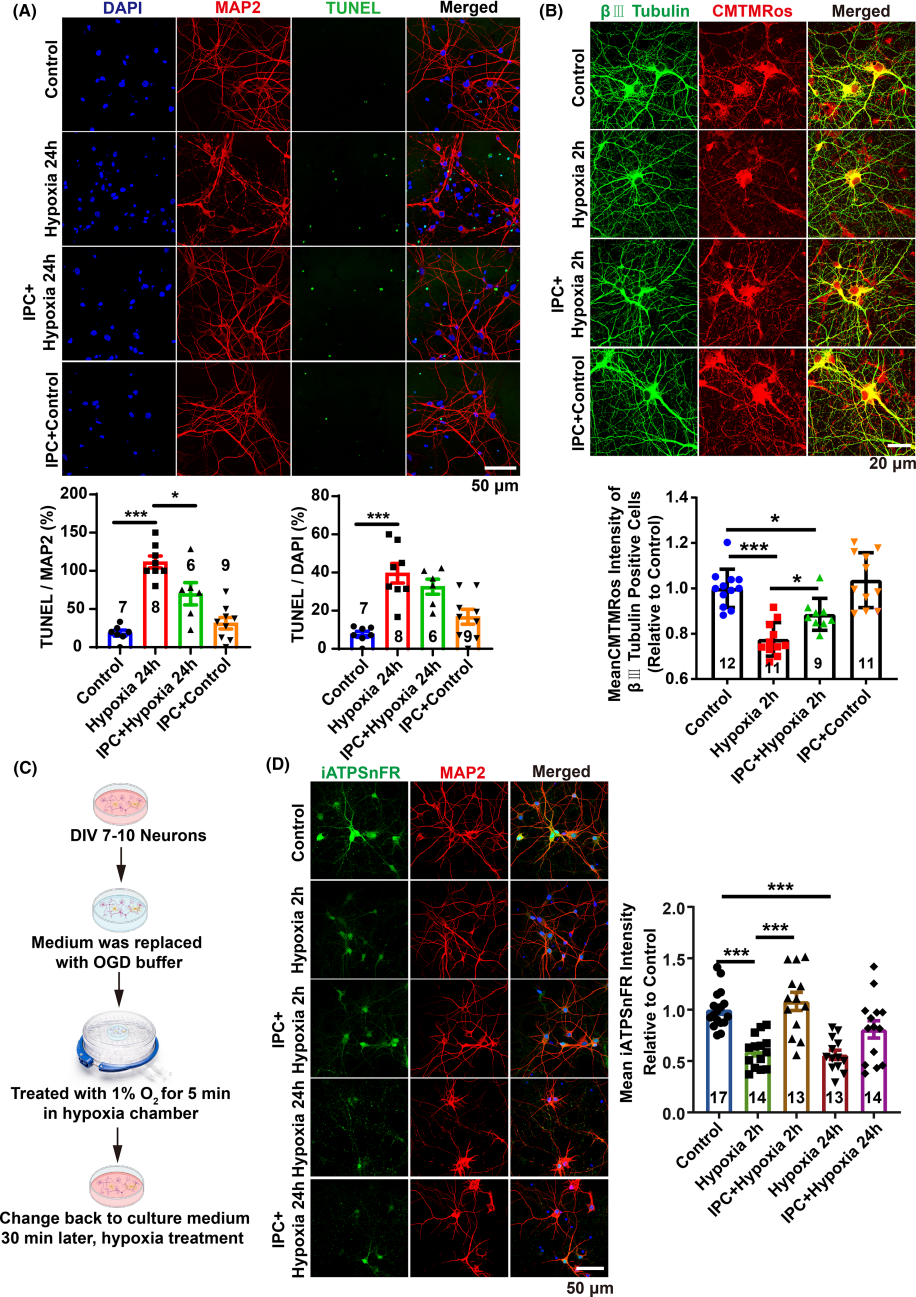
The protective effect of IPC on primary cortical neurons during hypoxia. (A) Images and quantitative analysis showing that cell apoptosis induced by 24 h hypoxia was rescued by IPC. (B) Images and quantitative analysis showing that mitochondrial membrane potential was rescued by IPC after 2 h hypoxia treatment. (C) Graphical diagram of IPC for primary cortical neurons. (D) Representative images and quantitative analysis showing that IPC attenuated the decrease in ATP levels induced by both 2 h and 24 h of hypoxia treatment, as compared to hypoxia alone. Scale bars: 50 μm (A, D), 20 μm (B). All data are presented as mean ± SD, with *n* = 3 biological replicates, and the total number of images for analysis is indicated within the bars. Statistical analysis was performed using the Shapiro–Wilk test for normality of all data, followed by one‐way ANOVA and Tukey's multiple comparisons test. *p < 0.05, ***p < 0.001.

Can strategies targeting glucose metabolic reprogramming and mitochondrial function protection serve as therapeutic interventions for cerebral edema? IPC has emerged as a clinically validated approach conferring neuroprotection and cardioprotection against ischemic and hypoxic stress.^17^, ^18^ The most commonly utilized in vitro model to simulate in vivo ischemia is oxygen–glucose deprivation (OGD).^28^ To evaluate the protective efficacy of IPC on neurons under hypoxic conditions in vitro, we employed a hypoxia chamber combined with glucose deprivation buffer to replicate ischemic preconditioning conditions observed in vivo (Figure 4C).

Our findings demonstrated that 24 h of hypoxia treatment resulted in abnormal neuronal morphology, as evidenced by fragmented MAP2 staining, and a significant increase in cell apoptosis, indicated by TUNEL labeling (Figure 4A). However, prior IPC treatment markedly reduced hypoxia‐induced cell apoptosis and preserved neuronal morphology (Figure 4A). Interestingly, IPC significantly ameliorated the reduction in mitochondrial membrane potential induced by 2 h of hypoxia (Figure 4B). Additionally, IPC treatment markedly elevated ATP levels within neurons after both 2 and 24 h of hypoxia treatment (Figure 4D). The results suggest that IPC may enhance mitochondrial function and promote oxidative metabolism, facilitating improved glucose metabolism and metabolic coupling with mitochondria. This ensures sufficient energy production under hypoxia conditions, thereby preventing the onset of cerebral edema due to energy depletion.

### 3.4 RIPC induces glucose metabolic reprogramming to elevate ATP levels in mitochondria

**FIGURE 5:**
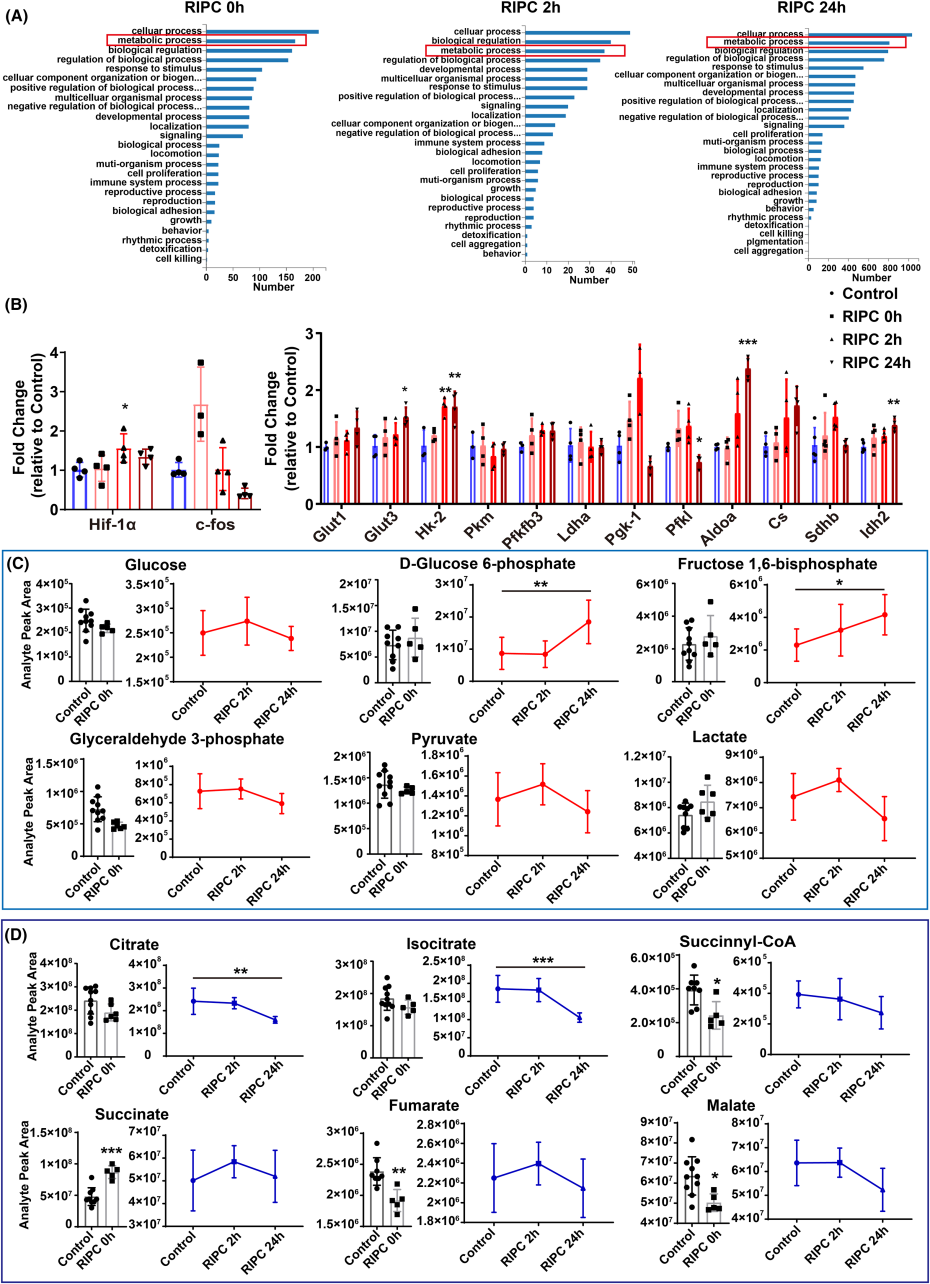
RIPC induced gene expression and metabolite changes involved in glucose metabolism. (A) Gene ontology analysis shows metabolic processes significantly regulated by RIPC at all three time points. (B) Validation of gene expression using real‐time PCR. (C,D) Variations in metabolites involved in glycolysis and the TCA cycle. Data are presented as mean ± SD, with *n* = 5–10 for each group. Statistical analysis: Shapiro–Wilk test for normality of all data; the one‐way ANOVA followed by Dunnett's multiple comparisons test for (B,C) and for citrate and isocitrate in (D); two‐sided unpaired *t*‐test between Control and RIPC 0 h for other metabolites in (D). *p < 0.05, **p < 0.01, ***p < 0.001.

To validate our hypothesis and elucidate the protective mechanisms of IPC, we conducted RNA sequencing and metabolomics assays on rat brain cortex at 0, 2, and 24 h post‐RIPC. We observed significant enrichment in metabolic processes across all three time points compared to other biological processes (Figure 5A). Quantitative real‐time PCR confirmed a substantial upregulation of hypoxia‐inducible factor HIF‐1α at 2 h post‐RIPC, known for initiating transcription of genes regulating extracellular glucose uptake and glycolytic enzymatic activity. Concurrently, expression levels of key metabolic genes including Hk‐2, Pfkfb3, Pgk‐1, Sdhb, and Idh2 were significantly elevated (Figure 5B).

Metabolomic analysis revealed increased levels of glucose, pyruvate, and lactate at 2 h post‐RIPC, alongside higher concentrations of glycolytic intermediates such as D‐glucose 6‐phosphate and fructose 1,6‐diphosphate at 24 h post‐RIPC (Figure 5C). Notably, TCA cycle intermediates, such as succinate, immediately increased, while succinyl‐CoA, fumarate, and malate decreased at RIPC 0 h (Figure 5D), demonstrating that RIPC promptly induced changes in the TCA cycle. At 2 h post‐RIPC, succinate and fumarate also showed an increase, while citrate and isocitrate decreased after hypoxia exposure (Figure 5D). These findings suggest that RIPC enhances glycolysis and simultaneously stimulate TCA cycle activity within 2 h post‐treatment, prior to the onset of brain edema.

**FIGURE 6:**
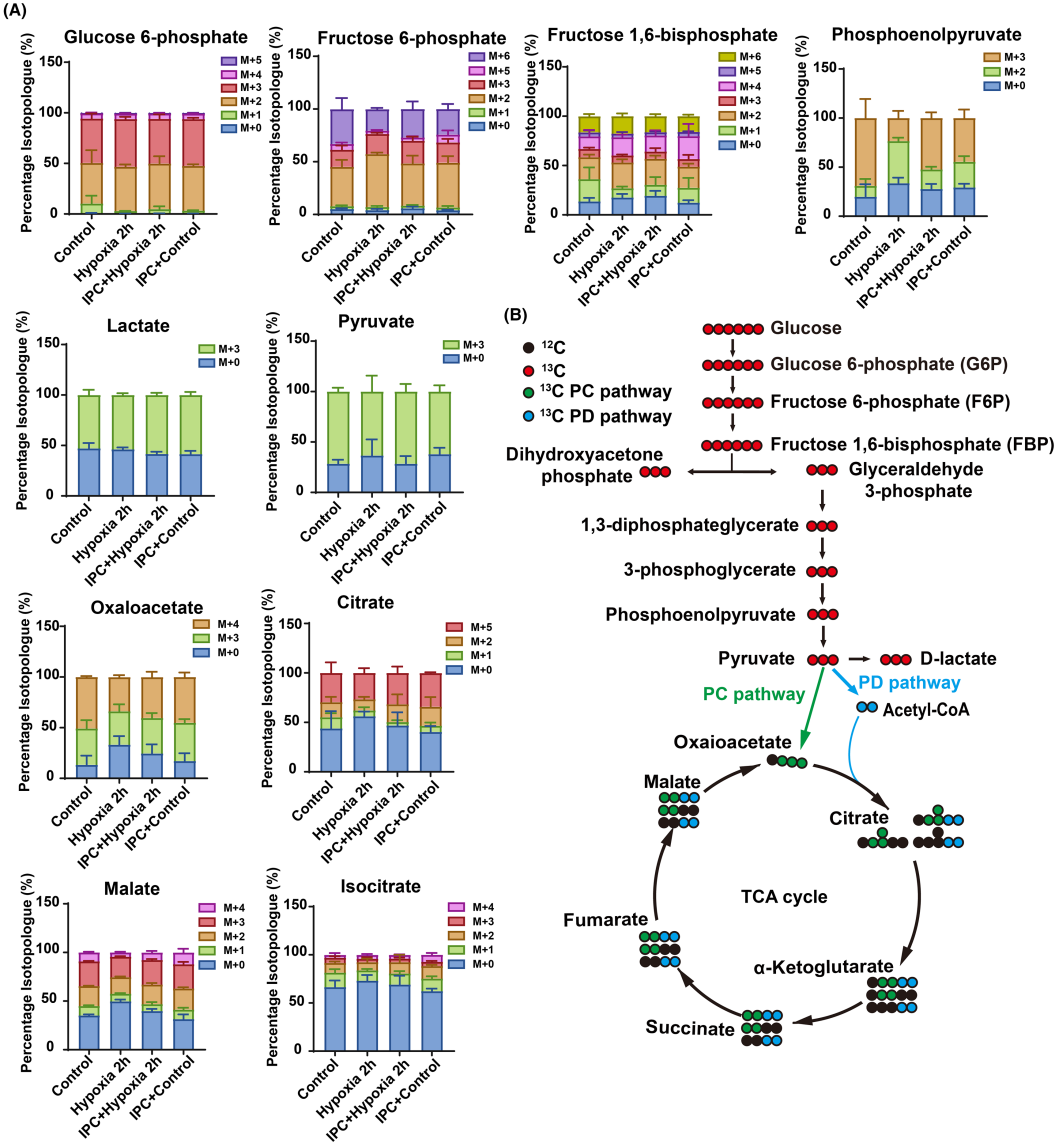
Promotive effect of IPC on mitochondrial ATP production during hypoxia. (A) Metabolic flux assay using isotope tracing experiments. (B) Representative image showing metabolic flux labeling. All data are presented as mean ± SD, *n* = 3 biological replicates.

As brain tissue consists of various cell types, we aimed to determine the direct impact of IPC on neuronal metabolism by tracing isotopes in purified rat cortical neurons exposed to ^13^C_6_‐U‐glucose. Compared to the hypoxia 2 h group, our findings revealed an increased isotope labeling ratio in the IPC + hypoxia group for key metabolites such as phosphoenolpyruvate, pyruvate, oxaloacetate, citrate, malate, and isocitrate (Figure 6A,B), suggesting that IPC facilitates glucose utilization within mitochondria under hypoxic conditions, aligning with the enhanced mitochondrial membrane potential for ATP production (Figure 4B,D). Together, these results demonstrate that IPC promotes mitochondrial ATP generation by augmenting mitochondrial glucose metabolism.

### 3.5 RIPC can prevent brain edema induced by high‐altitude in SD rats

**FIGURE 7:**
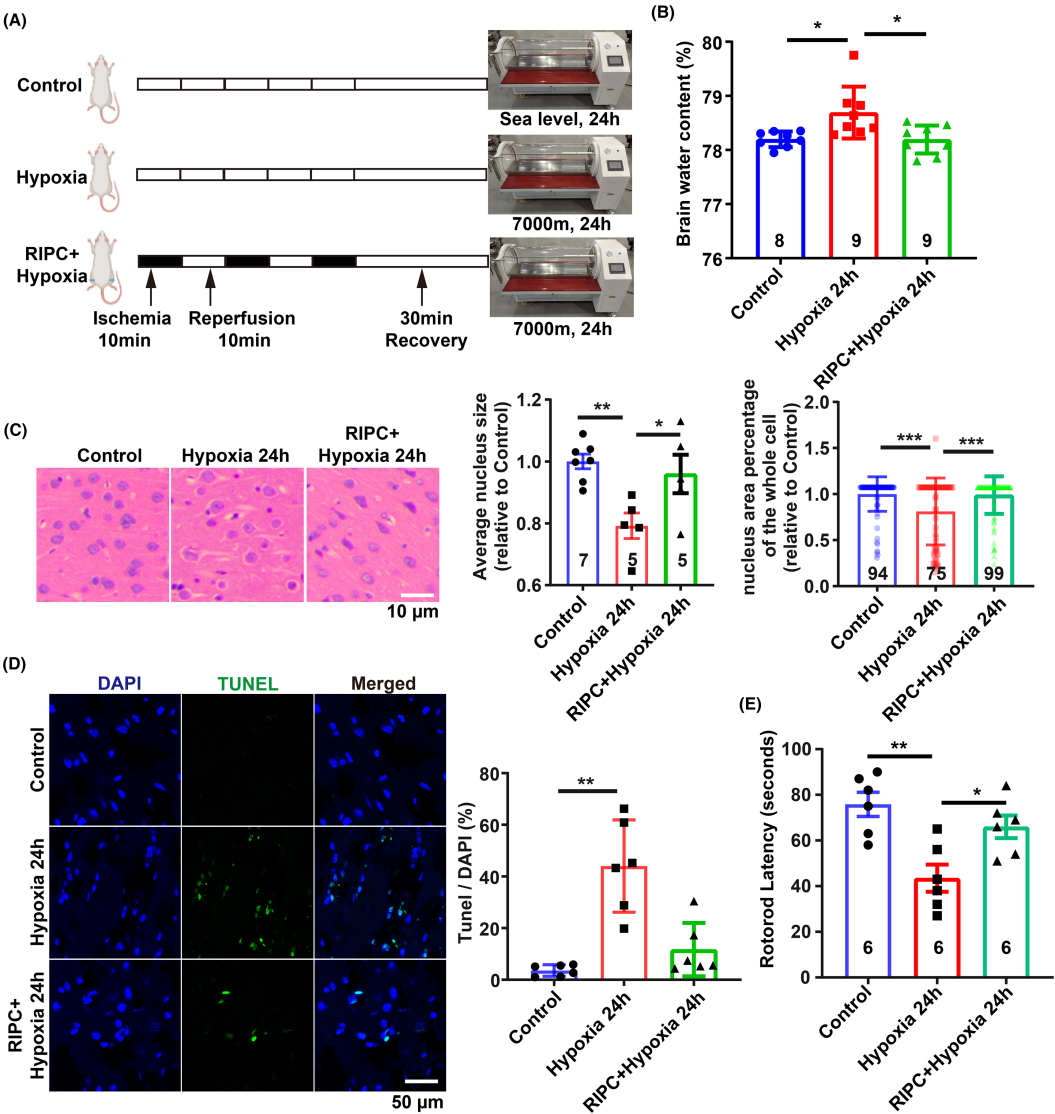
Protective effect of RIPC on brain edema induced by high‐altitude hypoxia. (A) Graphical diagram of RIPC on SD rats. (B) Brain water content assay showing RIPC significantly reduced brain water content induced by high‐altitude hypoxia. *n* = 8 or 9 mice per group. (C) Images of H&E staining and quantitative analysis showing RIPC significantly improved brain edema induced by high‐altitude hypoxia. (D) Images and quantitative analysis showing RIPC significantly reduced cell apoptosis induced by brain edema. (E) Quantitative analysis shows significant motor behavior improvement by RIPC after 24 h high‐altitude hypoxia treatment, with *n* = 6 mice per group. All data are presented as mean ± SD, with *n* = 3 biological replicates for (C,D), and the total number of images for analysis is indicated within the bars. Scale bar: 10 μm for (C) and 50 μm for (D). Statistical analysis was performed using the Kolmogorov–Smirnov test for normality of all data; one‐way ANOVA followed by Tukey's multiple comparisons test for (B,E), and for the average nucleus size in (C), and the Kruskal–Wallis test followed by Dunn's multiple comparisons test for (D) and for the nucleus area percentage in (C). *p < 0.05, **p < 0.01, ***p < 0.001.

To investigate the neuroprotective efficacy of RIPC in vivo, SD rats were subjected to high‐altitude hypoxia for 24 h (Figure 7A). Compared to the hypoxia‐only group, RIPC markedly mitigated cerebral edema, as evidenced by a significant reduction in brain water content (Figure 7B). Histopathological examination using H&E staining revealed that RIPC notably ameliorated hypoxia‐induced intracellular space enlargement (Figure 7C). Additionally, RIPC preconditioning led to a substantial decrease in cell apoptosis within the cerebral cortex (Figure 7D). Moreover, RIPC was associated with a significant improvement in motor function, as demonstrated by enhanced performance on the rotarod test, indicating improved neurological outcomes following cerebral edema (Figure 7E). Collectively, these findings highlight the therapeutic potential of RIPC in alleviating HACE and enhancing neural function.

## 4 DISCUSSION

In this study, we observed perturbations in glucose and energy metabolism during the progression of HACE and proposed that metabolic reprogramming strategies may compensate for energy deprivation and confer protection. We investigated the protective effects of RIPC against HACE and the underlying mechanisms. Our findings revealed that ischemic preconditioning on cells could boost glucose metabolism, coupled with mitochondrial energy production. We hypothesized that RIPC could improve outcomes in HACE by increasing ATP supply to compensate for the energy deprivation caused by hypoxia.

HACE manifests after the body is exposed to a low‐oxygen environment before acclimatization.^5^ As a serious high‐altitude illness, the pathophysiological mechanism of HACE remains unclear; early diagnosis is challenging, and effective prevention and treatment options are limited. It is hypothesized that the cerebral swelling in HACE stems from both vasogenic and cytotoxic edema. Vasogenic edema arises from the disruption of the blood–brain barrier (BBB) and subsequent fluid infiltration,^1^ while cytotoxic edema may result from cellular ion pump malfunction.^29^ A study found that ionic edema, a subtype of cerebral edema, also occurs in the brains of healthy individuals exposed to 16 h of normobaric hypoxia, mimicking a sudden ascent to a high altitude of 4500 meters.^30^ In addition, inflammatory activation may also be involved in HACE.^31^ Hypoxia‐activated microglia upregulate NRF1, which induces an inflammatory response by transcriptionally activating NF‐κB, p65, and mitochondrial transcription factor A. This process compromises BBB integrity and releases pro‐inflammatory factors, ultimately inducing HACE.^1^

Maintaining osmotic pressure within the BBB, neurons, and glial cells is an energetically demanding process. Disruption of the energy supply leads to water influx and consequent brain swelling. It has been established that hypoxia can deplete energy in cells, particularly in neurons,^32^, ^33^ suggesting that the primary etiology of HACE may be energy shortage. Under hypoxic conditions, mitochondrial hypoxia impairs normal oxidative phosphorylation and electron transport chain function, leading to oxidative phosphorylation dysfunction and the production of a large number of reactive oxygen species, which promote the occurrence of HACE.^34^ Our study established a rat model of HACE under hypobaric hypoxia conditions and applied transcriptomics and metabolomics to analyze the metabolic changes in the cerebral cortex associated with HACE. KEGG pathway enrichment analysis revealed dysregulation of glycolysis and oxidative phosphorylation in HACE, indicating energy depletion in the brains. In vitro, reduced ATP levels, diminished mitochondrial membrane potential, and aberrant mitochondrial morphology were observed, indicating significant functional impairment of mitochondria during hypoxia.^35^ Hence, enhancing energy production and mitochondrial function is a potential protection against HACE.

Given the similarities between hypoxia/ischemia and high‐altitude‐induced diseases, it is plausible to hypothesize that RIPC may protect against HACE. RIPC has recently emerged as an innovative therapeutic strategy in the prevention and treatment of cardiovascular and cerebrovascular diseases, distinct from conventional treatments.^16^, ^18^, ^36^ The mechanism of RIPC is complex and not fully understood, involving three primary mechanistic levels: remote stimulus, signal transfer (the neural and humoral pathway hypothesis), and targets and potential effectors.^37^ The humoral hypothesis proposes that a short‐term ischemic stimulus triggers local cells to produce substances that are released into the bloodstream during the reperfusion period and subsequently influence cells in remote organs, although specific humoral mediators remain completely unidentified.^37^ Our study used in vitro IPC models to simulate RIPC in vivo by directly subjecting cells to oxygen–glucose deprivation (OGD), integrating stimulation, signal transduction, and effector responses in neurons. This IPC model is supported by findings that mononuclear cells preconditioned by OGD in vitro secrete remodeling factors like vascular endothelial growth factor and transforming growth factor‐β, which have been found to be protective against cerebral ischemia.^38^ Although the complex neuro‐humoral interaction is not entirely clear and blocking neuronal signal transfer abrogates the protection provided by RIPC on remote target organs,^19^, ^37^ our IPC model counteracts the detrimental effects of hypoxia on neurons.

The RIPC stimulus triggers two distinct protective windows. The first window begins right after the stimulus, lasting for 2–3 h before diminishing. The second window emerges 12–24 h later and persists for 48–72 h.^39^, ^40^ Our study found that as early as 2 h after RIPC, the metabolic process was activated, and the hypoxia‐inducible factor‐1 (HIF‐1α) was markedly elevated, which is essential for RIPC.^41^, ^42^ This is in line with the study that in mice with HIF‐1α heterozygous knockout, the reduction in infarct size by RIPC was abrogated.^43^ HIF‐1α plays a critical role as a major regulator of metabolic enzymes in response to hypoxia, significantly impacting metabolism. This includes enhancing the efficiency of energy‐producing pathways by improving anaerobic glycolytic activity, as indicated by our results showing that the genes encoding enzymes involved in the glycolytic pathway are upregulated after RIPC. Additionally, HIF‐1α can directly target mitochondria to protect them from oxidative stress, preserve mitochondrial membrane potential, and reduce apoptosis.^44^ The discovery of small‐molecule HIF‐1α activators such as roxadustat and vadadustat^45^ holds the promise of pharmacological manipulation of HIF‐1α to simulate RIPC, promoting glycolysis and protecting mitochondrial function, thereby preventing the onset of HACE.

RIPC activates multiple intracellular signaling cascades that subsequently target intracellular pathways, notably metabolic pathways, to confer protection. It is well‐established that RIPC regulates the PI3K/Akt signaling pathway, leading to the activation of various downstream signaling elements.^36^ Ischemic preconditioning also reduces TSC2 phosphorylation at S1365, thereby increasing mTOR activation, promoting glucose utilization or glycolysis, and reducing mitochondrial fatty acid metabolism, which confers cardioprotection.^46^

Interestingly, most signal transduction pathways of RIPC converge on mitochondria, which are critical end effectors.^19^, ^37^ For survival of cardiomyocytes and myocardium salvage, RIPC is associated with preserved mitochondrial morphology and maintained mitochondrial membrane potential. RIPC inhibits mitochondrial permeability transition pore (mPTP) opening, which may otherwise initiate cell death through cytochrome C release and subsequent intracellular proteolysis.^47^ Additionally, RIPC induces mitochondrial ATP‐dependent K(+) channel opening in rat myocardium.^48^ However, how to couple cellular metabolism with mitochondria under hypoxia remains poorly understood. We revealed that IPC increases mitochondrial membrane potential and ATP production in neuronal mitochondria, likely corroborating glycolysis and mitochondrial coupling through a mitohormesis mechanism, thereby leaving the tissue in a less vulnerable state after stress.^49^ Such benefits are crucial for protecting mitochondrial function and sustaining ATP levels under hypoxic conditions. These findings indicated that the mechanisms of RIPC in preventing HACE may involve: (1) upregulating glycolysis, (2) inhibiting cell apoptosis, and (3) maintaining mitochondrial integrity and improving mitochondrial function. The enhanced integration of these processes allows for the rapid mobilization of metabolic reserves to adapt to hypoxic stress, which provides a protective effect of RIPC against HACE in a rat model, with significant improvements noted in brain edema and the associated motor dysfunction caused by cerebral edema.

Lastly, in this study, we cannot exclude the variation in gene expression and metabolite data obtained from cerebral cortex tissue that may reflect the results of brain injury and reactive responses involving numerous cell types. Additionally, the levels of glucose and lactate metabolites in brain tissue are directly influenced by their concentrations in the blood. However, our cell experiments directly identify the metabolic modulation and enhanced mitochondrial function in neurons after IPC, which helps counteract hypoxic damage. Nonetheless, RIPC has been shown to remodel the metabolism of the entire brain tissue, thereby alleviating cerebral edema. Furthermore, a study showed that hypoxia would be expected to reduce body temperature in rats,^50^ and the temperature appears to be a potentially important factor for metabolism. Hypoxia limits mitochondrial ATP production, which may contribute to a decrease in body temperature under hypoxic conditions. The impact of temperature on metabolism remains unclear.

## 5 CONSLUSION

In conclusion, our research detected glucose metabolic dysfunction during the development of HACE. RIPC played a role in stimulating glucose metabolism, down to the TCA cycle, to boost mitochondrial ATP production. Moreover, RIPC ameliorated brain edema and cellular apoptosis induced by high‐altitude hypoxia. Our findings support the potential of RIPC as a viable therapeutic approach for the prevention and treatment of HACE by targeting metabolic reprogramming.

## AUTHOR CONTRIBUTIONS

R.‐R.H. designed and executed the experiments, analyzed the data, and wrote the original manuscript; X.‐Y.Y. designed experiments, analyzed the data, and revised the manuscript; X.‐M.J. co‐designed and initiated the project; B.Z. is the senior author, designed and supervised the project.

## FUNDING INFORMATION

The author(s) disclosed receipt of the following financial support for the research, authorship, and/or publication of this article: This work was supported by Chinese Ministry of Science and Technology (2019YFA0508603), China; Beijing Municipal Natural Science Foundation (L222080 and 7192103); National Natural Science Foundation of China (822271513 and 81971198).

## CONFLICT OF INTEREST STATEMENT

The authors have no conflicts of interest to declare.

## Supporting information


Table S1.

## Data Availability

The data that support the findings of this study are available from the corresponding author upon request.
