# Supplementary material for: Remote ischemic preconditioning prevents high‐altitude cerebral edema by enhancing glucose metabolic reprogramming
Source: CNS Neurosci Ther. 2024 Sep 2;30(9):e70026. doi: 10.1111/cns.70026 (PMC11369019; doi:10.1111/cns.70026)
Supplement: Supplementary file 1 — Table S1. [file CNS-30-e70026-s001.docx]

**Supplement Table 1. Quantitative real-time PCR primer sequences**

| **Gene name** | **Forward (From 5’ to 3’)** | **Reverse (From 5’ to 3’)** |
| --- | --- | --- |
| Glut1 | CTCTGTCGGCCTCTTTGTTAAT | CCAGTTTGGAGAAGCCCATAAG |
| Glut3 | TCATCAATGCACCTGAGACAATC | GTCCCTCACTTGGTAGGTCTT |
| Hk-2 | TGATCGCCTGCTTATTCACGG | AACCGCCTAGAAATCTCCAGA |
| Pfkfb3 | CAACTCCCCAACCGTGATTGT | GAGGTAGCGAGTCAGCTTCTT |
| Pfkl | TTTTGGAGGTGATGGGACGG | TGTGGTTCTGGAGGCATCCTT |
| Pgk-1 | ATGTCGCTTTCCAACAAGCTG | GCTCCATTGTCCAAGCAGAAT |
| Pkm | GGTGGCTCTGGATACAAAGGG | ACTTCTCCATGTAAGCGTTGTC |
| Ldha | GATCTCGCGCACGCTACT | TGAATCTTTTGGGACCGCTT |
| Aldoa | CGTGTGAATCCCTGCATTGG | CAGCCCCTGGGTAGTTGTC |
| Cs | GGACAATTTTCCAACCAATCTGC | TCGGTTCATTCCCTCTGCATA |
| Sdhb | CTGAATAAGTGCGGACCTATGG | AGTATTGCCTCCGTTGATGTTC |
| Idh2 | GGAGAAGCCGGTAGTGGAGAT | GGTCTGGTCACGGTTTGGAA |
| Hif-1α | CACCAGACAGAGCAGGAAAGA | TGCTGCAGTAACGTTCCAATTCC |
| c-fos | GGCAGAAGGGGCAAAGTAGA | AGTTGATCTGTCTCCGCTTGG |
| Actin | CGTCCACCCGCGAGTACAA | ATGGCGAACTGGTGGCG |
